# Sishun Formula for acute migraine attack: study protocol for a double-blind, randomized, placebo-controlled trial

**DOI:** 10.3389/fneur.2025.1643130

**Published:** 2025-11-06

**Authors:** Xuran Zhang, Jiaojiao Liu, Huanqin Li, Bo Zhou, Shaoqing Wang, Lexi Li, Xuefeng Wu, Kegang Cao

**Affiliations:** 1 Dongzhimen Hospital, Beijing University of Chinese Medicine, Beijing, China; 2 Beijing Tiantan Hospital Affiliated to Capital Medical University, Beijing, China; 3 Xiyuan Hospital of China Academy of Chinese Medical Sciences, Beijing, China

**Keywords:** Sishun Formula, acute migraine attack, randomized controlled trial, study protocol, traditional Chinese medicine

## Abstract

**Purpose:**

Migraine, a leading global cause of disability, affects over 1.1 billion individuals worldwide. Current acute pharmacotherapy is limited by contraindications and adverse events (AEs), underscoring the need for safer, more effective alternatives. The Sishun Formula (SSF), a traditional Chinese medicine (TCM) formulation targeting “Ying-Wei disharmony,” demonstrated promising preclinical and preliminary clinical results. This trial aims to rigorously evaluate SSF’s efficacy and safety for acute migraine attacks.

**Patients and methods:**

This multicenter, randomized, double-blind, placebo-controlled trial will enroll 144 participants with acute migraine and “Ying-Wei disharmony” syndrome, randomized 1:1 to SSF or placebo. A single dose of SSF or a matched placebo is administered within 0.5 h of migraine onset. The primary outcome is the proportion achieving headache freedom at 2 h post-dose. Secondary outcomes include the proportion achieving headache relief at 2 h post-dose, absence of most bothersome associated symptoms, sustained relief/freedom at 24/48 h, the Visual Analogue Scale (VAS) score changes, time to efficacy onset, recurrence rates, and TCM syndrome improvement. Biomarkers [e.g., calcitonin gene-related peptide (CGRP)] and safety parameters (vital signs, laboratory tests, AEs) are monitored. Data analysis will employ SPSS 25.0 and R 4.3.1.

**Conclusion:**

The findings are anticipated to deliver high-quality evidence validating SSF’s efficacy and safety, offering a promising therapeutic alternative for acute migraine attacks.

**Clinical trial registration:**

http://itmctr.ccebtcm.org.cn/, identifier ITMCTR2025000187.

## 1 Introduction

Migraine ranks as the 3rd leading cause of disability-adjusted life-years (DALYs) among all nervous system disorders globally, and the top cause of DALYs in adolescents (5–19 years). It affects 14.9% of the global population (1.16 billion individuals), making it the most prevalent neurological condition by prevalence (1). Beyond its hallmark pain, the acute migraine attack is often associated with photophobia, phonophobia, nausea, and vomiting. These symptoms exacerbate disability, disrupt daily activities, and elevate risks of comorbid conditions such as anxiety, depression, and cardiovascular disorders (2). Guidelines indicate that acetaminophen, acetylsalicylic acid, ibuprofen, naproxen sodium, and triptans have the highest level of evidence for the treatment of acute migraine attacks (3, 4). In recent years, gepants have emerged as novel calcitonin gene-related peptide (CGRP) receptor antagonists offering a favorable safety profile for acute migraine treatment, particularly in patients with contraindications (5). Despite the availability of these medications, many patients experience inadequate relief from their symptoms (6). It was reported that a significant proportion of migraine sufferers (60–88%) failed to achieve pain freedom at 2 h after taking the triptans (7). Additionally, frequent use of these medications may lead to adverse events (AEs) such as medication-overuse headaches, gastrointestinal complications, and cardiovascular events (6, 8, 9), underscoring the urgent need for safer, more effective alternatives.

Traditional Chinese medicine (TCM) attributes migraine pathogenesis to imbalances in Ying and Wei, collectively termed “Ying-Wei disharmony” (10). According to TCM theory, Ying (nutritive Qi) and Wei (defensive Qi) govern physiological harmony, with their disharmony manifesting as disordered Qi-blood circulation and compromised visceral function, ultimately triggering migraine (11). In modern medicine, Ying and Wei are recognized as essential substances responsible for maintaining homeostasis of the vascular intima and adventitia, as well as balancing energy metabolism and immune defense (12). Ying-Wei imbalance is associated with key pathological mechanisms of migraine, including dysregulation of neuroinflammatory pathways, mitochondrial metabolic dysfunction, autonomic nervous system dysfunction, and impaired vasomotor activity (12).

Based on this theoretical framework and incorporating the extensive clinical expertise of TCM specialist Kegang Cao, we developed the Sishun Formula (SSF) (13). It is a pharmacologically optimized compound comprising five medicinal botanicals (constituent details provided in Table 1), each with documented analgesic and anti-inflammatory properties as evidenced by modern studies (14–18). Our preclinical studies in migraine-model rats demonstrated that SSF significantly reduced serum and trigeminal ganglion (TG) levels of CGRP while elevating 5-hydroxytryptamine (5-HT) concentrations, with concomitant downregulation of nuclear factor-kappa B (NF-κB) and cyclooxygenase-2 (COX-2) protein expression in TG tissues, thereby ameliorating neurogenic inflammation (19).

**Table 1:** The Chinese herbal components and dose of SSF.

Chinese name	Botanical nomenclature with taxonomic classification	Dosage (g)	Processed botanical material
Guizhi	*Cinnamomum cassia* (L.) J. Presl (Lauraceae)	10	Dried twig
Baishao	*Paeonia lactiflora* Pall. (Paeoniaceae)	10	Dried root
Chuanxiong	Conioselinum anthriscoides “Chuanxiong” (Apiaceae)	10	Dried root and rhizome
Niuxi	*Achyranthes bidentata* Blume (Amaranthaceae)	10	Dried root
Hongjingtian	Rhodiola crenulata (Hook.f. & Thomson) H.Ohba (Crassulaceae)	6	Dried root and rhizome

However, robust clinical validation through randomized controlled trials (RCTs) remains absent, limiting its integration into evidence-based practice. Therefore, this study will implement a multicenter, randomized, double-blind, placebo-controlled trial to further evaluate the efficacy and safety of SSF for acute migraine attacks. By employing the internationally recognized efficacy endpoints and integrating dynamic monitoring of neuropeptide biomarkers, we aim to elucidate the therapeutic mechanisms of SSF within a modern research framework and provide a safe and effective alternative option for migraineurs.

## 2 Methods and analysis

### 2.1 Formulation preparation

SSF, classified as a Type A botanical extract under pharmacopoeial standards (20), is a phytopharmaceutical formulation comprising five pharmacologically standardized botanical drugs processed through modern pharmaceutical techniques, including decoction, concentration, drying, and granulation (Supplementary material 1). The formula integrates *Cinnamomum cassia* (L.) J. Presl (Lauraceae; Guizhi-dried twig) (10 g), *Paeonia lactiflora* Pall. (Paeoniaceae; Baishao-dried root) (10 g), *Conioselinum anthriscoides* “Chuanxiong” (Apiaceae; Chuanxiong-dried root and rhizome) (10 g), *Achyranthes bidentata* Blume (Amaranthaceae; Niuxi-dried root) (10 g), and *Rhodiola crenulata* (Hook.f & Thomson) H.Ohba (Crassulaceae; Hongjingtian-dried root and rhizome) (6 g), harmonizing to regulate Ying, Wei, Qi, and Blood, and is therefore named “Sishun.” Quality control protocols employ high-performance liquid chromatography (HPLC) to validate botanical authenticity, quantify bioactive markers, and ensure batch-to-batch consistency, adhering to standard pharmacopoeial specifications for identity, purity, and extraction efficiency (21). Dosage parameters align with evidence-based clinical practices, utilizing processed botanical materials standardized to their pharmacologically active forms for optimized therapeutic delivery. The Chinese herbal components and dose of SSF are presented in Table 1.

### 2.2 Trial design

This is a multicenter, randomized, double-blind, placebo-controlled, single-attack clinical trial conducted across three centers: Dongzhimen Hospital affiliated to Beijing University of Chinese Medicine, Xiyuan Hospital of China Academy of Chinese Medical Sciences, and Beijing Tiantan Hospital affiliated to Capital Medical University. A total of 144 eligible participants will be randomized in a 1:1 ratio to either the experimental group (SSF) or the control group (placebo). The study comprises a 4-week screening period, a 45-day intervention period, and a 7-day follow-up period. During the intervention phase, participants will administer SSF or placebo upon experiencing an eligible acute migraine attack, with efficacy and safety assessments conducted at the pre-dose time point after headache onset, 0.5 h, 1.0 h, 2.0 h, 4.0 h, 24 h, and 48 h post-dose. An eligible acute migraine attack must meet two criteria: (1) No prior use of migraine-specific medications (including all Western pharmaceuticals, TCM formulations in any dosage form, or TCM patent medicines explicitly approved for migraine treatment in their prescribing information) within the 24-h period preceding the current episode; (2) The current migraine episode is of moderate to severe intensity [Visual Analogue Scale (VAS) ≥4 scores].

**Figure 1:**
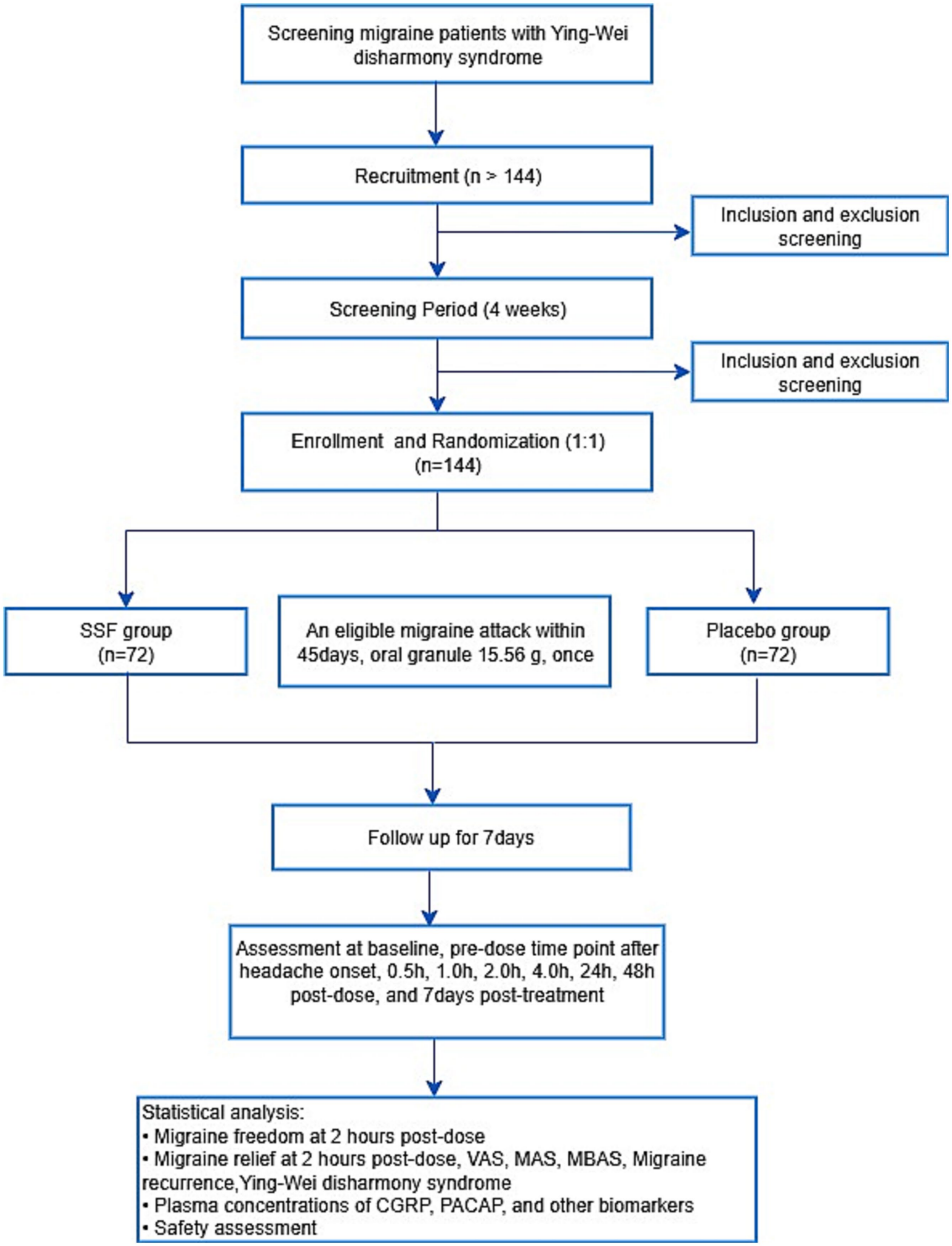
Study flowchart. SSF, Sishun Formula; VAS, Visual Analogue Scale; MAS, migraine-associated symptom; MBAS, most bothersome associated symptom; CGRP, calcitonin gene-related peptide; PACAP, pituitary adenylate cyclase-activating polypeptide.

The protocol adheres to the Standard Protocol Items: Recommendations for Interventional Trials (SPIRIT) 2025 guideline (22). The study flowchart is provided in Figure 1, with enrollment, intervention, and assessment timelines detailed in Table 2.

**Table 2:** SPIRIT 2025 participant timeline: schedule of enrollment, interventions, and assessments.

Items	Trial period							
	Screening period	Baseline period	Intervention period (waiting for an eligible migraine attack within 45 days)					Follow-up period
Timepoint	−5 to −1 weeks	−1 to 0 weeks	Within 0.5 h of headache onset	Pre-dose time point after headache onset	0.5 h, 1.0 h post-dose	2.0 h, 4.0 h, 24 h post-dose	48 h post-dose	7 days post-dose
Enrollment								
Eligibility screen	X	X						
Informed consent		X						
Randomization		X						
Intervention								
Sishun Formula, once			X					
Placebo, once			X					
Information collection								
Demographic data	X							
Medication history	X	X						
Migraine episode characterization	X	X						X
Efficacy assessment								
VAS score				X	X	X	X	
Migraine-associated symptoms				X		X	X	
Most bothersome associated symptom				X		X	X	
Migraine recurrence						X	X	
Ying-Wei disharmony syndrome score		X					X	X
Acute analgesic utilization				X	X	X	X	
Plasma CGRP, PACAP, and other indicators		X					X	
Safety assessment								
Complete blood count		X					X	
Urinalysis		X					X	
Liver and kidney function test		X					X	
ECG		X					X	
AE					X	X	X	X
SAE					X	X	X	X

h, hour; VAS, Visual Analogue Scale; CGRP, calcitonin gene-related peptide; PACAP, pituitary adenylate cyclase-activating polypeptide; ECG, electrocardiogram; AE, adverse event; SAE, serious adverse event.

“X” indicates required procedures at designated time points.

### 2.3 Participants

#### 2.3.1 Diagnostic criteria for migraine

According to the diagnostic criteria for “migraine without aura” and “migraine with typical aura” outlined in the International Classification of Headache Disorders, 3rd edition (ICHD-3) (23).

#### 2.3.2 Diagnostic criteria for “Ying-Wei disharmony” syndrome

This syndrome is characterized by the presence of at least one of the following clinical features: (1) aversion to wind; (2) spontaneous sweating; (3) cold intolerance or cold extremities; (4) mental fatigue; and (5) palpitations. The differentiation of the syndrome will be independently assessed by two qualified researchers.

#### 2.3.3 Inclusion criteria

Eligible participants must fulfill all the criteria listed below:

1. Patients who both meet the diagnostic criteria for migraine and “Ying-Wei disharmony” syndrome.
2. Patients who are aged 18–65 years (inclusive).
3. Patients whose first migraine episode occurred before the age of 50.
4. Patients who have a documented migraine history of at least 1 year.
5. Patients who experience 2 to 8 moderate-to-severe migraine attacks (VAS ≥4 scores) within the past 4 weeks, with a minimum interval of 48 h between consecutive episodes.
6. Patients must be able to distinguish migraine from other types of headaches, such as tension-type headaches.
7. Patients who voluntarily participate in the study with written informed consent provided by the participant or their legal guardian.

#### 2.3.4 Exclusion criteria

Participants meeting any of the following criteria will be excluded:

1. Patients with ≥15 migraine days within the past 4 weeks.
2. Patients who have used analgesics for ≥10 days within the past 4 weeks.
3. Patients with secondary headaches attributed to organic pathologies, including but not limited to subarachnoid hemorrhage, cerebral hemorrhage, cerebral infarction, vascular malformations, arteritis, hypertension, or atherosclerosis.
4. Patients with severe circulatory, digestive, respiratory, or hematopoietic system diseases or cancer.
5. Patients with a history of epilepsy, stroke, other neurological disorders, or psychiatric conditions.
6. Patients with severe liver diseases, or those presenting with abnormal liver function, defined as aspartate aminotransferase (AST) or alanine aminotransferase (ALT) levels greater than 1.5 times the upper limit of the normal range (ULN).
7. Patients with severe kidney diseases, or those presenting with abnormal kidney function, defined as creatinine (Cr) levels exceeding the ULN.
8. Patients exhibiting significant anxiety or depression symptoms [Hamilton Anxiety Rating Scale (HAMA) ≥15 scores or Hamilton Depression Rating Scale (HAMD) ≥18 scores].
9. Patients with cognitive impairment, manifested as an inability to read or comprehend assessment scales, non-compliance with medical treatment, or inability to complete efficacy evaluations.
10. Patients using migraine prophylaxis at stable doses for <3 months.
11. Patients who are pregnant, lactating, or planning pregnancy.
12. Patients currently participating in other clinical trials.

#### 2.3.5 Withdrawal/termination criteria

Participants will be discontinued from the study if any of the following criteria are met:

1. Patients who voluntarily request to discontinue their participation in the study.
2. Patients who are unable to follow the study procedures or become lost to follow-up during the trial period.
3. Patients who experience serious AEs.
4. Patients with pregnancy detected after trial enrollment.
5. Patients demonstrating absence of an eligible acute migraine attack within 45 days after enrollment.

### 2.4 Randomization and allocation

The randomization scheme was designed and managed by an independent statistical unit using block randomization methodology. A random allocation sequence was generated through the PROC PLAN procedure in the Statistical Analysis System (SAS) V9.4 software. The randomization procedure was supervised by statisticians from the Good Clinical Practice (GCP) Center at Dongzhimen Hospital, Beijing University of Chinese Medicine. Critical parameters, including the selected block size and randomization table, were sealed and stored at the GCP center of Dongzhimen Hospital. Study medication allocation across trial sites commenced with the lowest assigned identifier, with eligible participants receiving drugs sequentially according to enrollment chronology.

### 2.5 Blinding

Blinding was administered by an independent statistical unit without direct involvement in trial operations. The team reviewed the trial medications and placebos to ensure their consistency in appearance and odor. Subsequently, the team standardized the drugs’ packaging and labeling configurations, with preassigned randomization codes affixed to all pharmaceutical containers. The blinding protocols were thoroughly documented and archived under restricted access. Emergency unblinding is permitted when investigators deem immediate intervention imperative for participant safety. However, the reasons for unblinding, the date of the procedure, and the signature of the site principal investigator must be fully documented.

### 2.6 Intervention

A single dose of granule therapy is to be administered to both groups within 0.5 h following the onset of an eligible migraine attack. Patients in the treatment group are orally administered SSF granules (2 bags at 7.78 g each, with warm water) within 0.5 h of headache onset. Patients in the control group are orally administered placebo granules (2 bags at 7.78 g each, with warm water) within 0.5 h of headache onset. Both SSF and placebo granules were produced by Sichuan Neo-Green Pharmaceutical Technology Development Company Limited. The placebo was formulated with maltodextrin, coloring agents, bittering agents, and 5% of the SSF herbs to sensorily match the active granules in appearance, taste, and odor, thereby protecting blinding integrity while ensuring negligible pharmacological activity.

Additionally, researchers will provide migraine management counseling to both groups. During the clinical trial period, patients may utilize alternative acute analgesic treatments for headache episodes that do not meet eligibility criteria, provided they contact researchers for treatment registration. In cases where the trial drugs fail to alleviate symptoms during an eligible migraine attack (i.e., no improvement in headache severity, impairments in daily or occupational functioning, or mood alterations), researchers will maintain open communication with patients, permit the use of conventional Western acute analgesics under supervision, and provide guidance on medication administration and post-intervention monitoring. Participants will also be instructed to record the timing and type of rescue medication used.

### 2.7 Outcomes and measurements

Efficacy evaluation is conducted through a specifically designed migraine patient-reported form. Patients are instructed to document migraine severity and migraine-associated symptoms (MASs) at pre-dose and at 0.5, 1, 2, 4, 24, and 48 h post-dose (24). To ensure data accuracy, physicians will review all entries in the form. In cases of implausible or inconsistent data, the attending physician will further inquire with the patient to verify the reported information. Pain intensity is quantified using the VAS and categorized into four severity levels: 0 (no pain), 1–3 (mild pain), 4–6 (moderate pain), and 7–10 (severe pain). To ensure reliable self-assessment during migraine attacks, all participants receive structured pre-trial training on the use of the VAS. This training includes standardized verbal instructions and practical examples to enhance comprehension and consistency in real-time reporting. Additionally, a simplified VAS guide emphasizing anchor points (e.g., “0 = no pain,” “10 = worst imaginable pain”) is provided for quick reference. The MASs, including photophobia, phonophobia, nausea, and vomiting, are assessed via a binary scale (0 for absence; 1 for presence) (25). Detailed outcome measurements at each time point are summarized in Table 2.

#### 2.7.1 Primary outcome

The primary endpoint is the proportion of patients achieving headache freedom at 2 h post-dose. Headache freedom is defined as the reduction in headache severity from baseline moderate or severe to no pain, as measured by the VAS.

#### 2.7.2 Secondary outcomes

1. The proportion of patients achieving absence of the most bothersome associated symptom (MBAS) at 2 h post-dose. Patients will self-identify their MBAS from the following symptoms at migraine onset: photophobia, phonophobia, nausea, or vomiting.
2. The proportion of patients achieving headache relief at 2 h post-dose. Headache relief is defined as the reduction of initial pain intensity from moderate to severe levels down to mild or no intensity.
3. The proportion of patients sustaining headache relief from 2 to 24/48 h post-dose. Sustained headache relief from 2 to 24/48 h is defined as achieving headache relief without rescue medication use and the absence of moderate-to-severe headache recurrence during 2 to 24/48 h following initial treatment.
4. The proportion of patients sustaining headache freedom from 2 to 24/48 h post-dose. Sustained headache freedom from 2 to 24/48 h is defined as achieving headache freedom without rescue medication use, coupled with the absence of any headache recurrence (mild, moderate, or severe) during 2 to 24/48 h following initial treatment.
5. Change in VAS scores from pre-dose to 0.5, 1, 2, 4, 24, and 48 h post-dose.
6. Time to onset of efficacy, defined as the interval (in minutes) between drug administration and headache relief.
7. Headache recurrence rate between 2 and 48 h post-dose. Headache recurrence is defined as patients who achieved headache relief (freedom) at 2 h post-dose, reexperiencing moderate-to-severe headache (headache of any severity) within the 2 to 48 h post-dose window.
8. Change in TCM “Ying-Wei disharmony syndrome.” The syndrome will be assessed using the Ying-Wei Disharmony Assessment Scale (YWDAS) at baseline, 48 h post-dose, and 7 days post-dose. The scale comprises five core TCM syndromes, and the assessment framework is detailed in Table 3. Its development was grounded in clinical expertise from senior TCM practitioners and integrated classical and contemporary medical literature. Content validity was established through a rigorous review and consensus by a panel of five senior TCM experts. In a pilot study to assess feasibility and inter-rater reliability, all YWDAS assessments were independently performed by two TCM researchers who underwent standardized training using predefined scoring criteria to minimize assessment bias. The inter-rater reliability, assessed by the intraclass correlation coefficient (ICC) for absolute agreement, was 0.76 (95% CI: 0.64–0.84), indicating good agreement.

**Table 3:** The Ying-Wei Disharmony Assessment Scale.

Symptom	None (0 point)	Mild (1 point)	Moderate (2 points)	Severe (3 points)	Score
Aversion to wind	Absent	Marked aversion when exposed to the draft	Marked aversion, even without wind exposure	Severe aversion unrelieved by added clothing	
Spontaneous sweating	Absent	Occasional hyperhidrosis or marked post-exercise sweating	Frequent sweating during daily activities	Frequent hyperhidrosis unrelated to physical exertion	
Cold intolerance	Absent	Subjective cold sensitivity	Subjective cold sensitivity and cold extremities	Cold extremities unrelieved by added clothing	
Palpitations	Absent	Exercise/effort-induced, relieved by rest	Frequent occurrence, relieved by rest	Frequent occurrence, unrelieved by rest	
Mental fatigue	Absent	Occasional fatigue, minimal work/study impact	Frequent fatigue with work/study impairment	Debilitating fatigue with significant functional decline	

#### 2.7.3 Exploratory indicators

We will assess changes in plasma concentrations of CGRP, PACAP, and other biomarkers. Blood samples will be collected at two specific timepoints: baseline and post-treatment. Serum levels of CGRP and PACAP will be quantified using enzyme-linked immunosorbent assays (ELISAs) that have been rigorously validated for clinical trial applications, ensuring reliability, sensitivity, and specificity. Furthermore, correlation analyses will be conducted to evaluate the association between changes in biomarker levels and clinical outcomes, particularly migraine intensity.

### 2.8 Safety assessments

Safety assessments involve the systematic monitoring of AEs and serious adverse events (SAEs), along with the evaluation of vital signs (including respiratory rate, heart rate, radial pulse, and blood pressure), routine laboratory parameters (such as complete blood count, urinalysis, liver and kidney function tests), and electrocardiograms (ECGs). All assessments are conducted at baseline and 48 h post-dose.

### 2.9 Sample size and recruitment

The sample size was calculated based on the primary outcome measure (the proportion of patients achieving headache freedom at 2 h post-dose). Sample size determination derived from the treatment effect of 35% versus 11% placebo response (based on unpublished data), calculated through binomial proportion comparison with one-sided *α* = 0.025, *β* = 0.1, and a 24% superiority margin. This calculation yielded a requirement of 60 participants per arm. To accommodate clinical enrollment uncertainties, including anticipated attrition and protocol deviations, the adjusted enrollment target was set at 144 participants, with 72 in each group. Patient recruitment will be conducted through neurology outpatient clinics and poster-based advertising, leveraging the high patient volume across three centers to ensure sufficient enrollment.

### 2.10 Data management

Data management is conducted using the Yidao Research Platform to ensure systematic oversight. Two trained researchers are responsible for entering research data accurately at specified follow-up intervals across all study centers. A comprehensive data cleaning process will be implemented, encompassing automated system checks and manual validation to identify inconsistencies, followed by iterative query resolution between data managers and investigators until all discrepancies are reconciled. Prior to database closure, a formal blind review will be conducted, with the data manager submitting a closure request accompanied by a blind review report and a database closure checklist. This checklist will confirm the completion of six critical criteria: full incorporation of all collected data (including resolved AEs), resolution of outstanding queries, verification of coding consistency, final review of anomalous data, archival compliance with standard operating procedures, and signed approval of the blind review report. Database closure will require documented authorization from stakeholders, after which editing permissions are revoked, with the locking timestamp serving as audit evidence. Post-closure errors will be rigorously documented and assessed for impact on safety/efficacy analyses through multidisciplinary consultation. Database modifications post-lock will require formal approval and restricted access, with relocking procedures mirroring initial protocols. Data integrity is further safeguarded through dual-entry validation, pre-processing audits for missing values and logical errors, and secure archival of source documents by the primary research unit, ensuring full traceability of all corrections via dated, initialed annotations.

### 2.11 Statistical analysis

All statistical analyses will be performed using SPSS 25.0 and R 4.3.1 software. Baseline characteristics will be presented through descriptive statistics [mean ± standard deviation or median (interquartile range) or frequency (percentage)]. For between-group balance testing, independent samples *t*-tests will be employed for normally distributed data with homogeneity of variance, while Mann–Whitney *U* tests are used otherwise. Categorical variables will be analyzed using chi-square tests or Fisher’s exact test based on expected frequencies. All predetermined potential confounders will be included as covariates in subsequent models for adjustment, regardless of their between-group balance status. Additionally, baseline variables showing significant imbalance will undergo sensitivity analyses to assess their confounding effects.

Efficacy analyses will be performed on both intention-to-treat (ITT) and per-protocol (PP) populations, with the ITT set including all randomized participants analyzed according to their allocated groups, regardless of protocol deviations, while the PP set will consist of participants who complied with the trial procedures without major deviations. For efficacy assessment, the use of rescue medication before the 2-h time point will be considered a treatment failure for the primary and key secondary endpoints (e.g., headache freedom, headache relief, absence of most bothersome symptom) (26). Accordingly, participants will be included in the 2-h efficacy analysis only if they have not used rescue medication before that time point, even if medication is taken afterward. Similarly, the assessment of sustained outcomes (e.g., sustained headache freedom at 24/48 h) will require no use of rescue medication within the respective 24/48-h timeframe.

The primary efficacy endpoint (2-h headache freedom) will be compared between groups using the chi-square test or Fisher’s exact test, with reporting of risk difference (RD) and relative risk (RR) accompanied by 95% confidence intervals (CIs). For secondary endpoints, categorical variables will be analyzed through logistic regression with baseline covariate adjustment, while continuous variables (e.g., VAS scores) will be assessed using a mixed-effects model for repeated measures (MMRM). Time to onset of efficacy will employ Kaplan–Meier curves with the Log-rank test and Cox proportional hazards models to estimate hazard ratios. To address missing data, multiple imputation will be employed under the assumption of missing at random, using fully conditional specification with predictive mean matching for continuous variables and logistic regression for categorical variables, incorporating baseline and post-baseline covariates; 20 imputed datasets will be generated, and results will be pooled using Rubin’s rules. To control for multiplicity, secondary endpoints will be grouped *a priori* into clinically coherent families, each tested using the Holm–Bonferroni method to control the family-wise error rate. The predefined families are: (1) a 2-h symptom response family comprising absence of the most bothersome symptom and headache relief at 2 h; (2) a sustained efficacy family consisting of sustained headache relief and sustained headache freedom from 2 to 24/48 h; and (3) a time-to-event and recurrence family including time to onset of efficacy and headache recurrence rate. Changes in VAS scores and the TCM syndrome score will be analyzed exploratorily and are not included in the adjustment. Safety analyses will be conducted in the safety set (SS), which includes all randomized participants receiving at least one dose of the study medication. This population will be summarized using descriptive statistics for adverse events, vital signs, and laboratory parameters, with between-group comparisons following the methods used for baseline characteristics.

### 2.12 Patient and public involvement statement

In the early stage of protocol development, structured surveys were administered to migraine patients to assess their therapeutic needs and preferences regarding TCM for acute pain management. Patients or the public are not involved in the conduct, reporting, or dissemination plans of our study.

## 3 Discussion

Migraine, a leading cause of global disability, profoundly impacts patients’ quality of life and socioeconomic productivity due to its debilitating symptoms and high recurrence rates (27, 28). Migraine attacks involve a complex interplay of multiple pathophysiological mechanisms, including aberrant neuronal hyperexcitability, neurogenic inflammatory responses, and subsequent central sensitization, ultimately culminating in severe cephalalgia and associated symptoms (29, 30). Current first-line therapies, including triptans and non-steroidal anti-inflammatory drugs (NSAIDs), do not always yield satisfactory outcomes, and prolonged use of these medications may precipitate AEs such as medication-overuse headache and cardiovascular complications (31–33).

TCM, with its holistic approach and emphasis on restoring physiological harmony, offers a promising therapeutic avenue for migraine, and its clinical efficacy has been validated by numerous studies (34, 35). Emerging evidence indicates that certain TCM formulations and herbal extracts exert multimodal effects, including inhibition of neurogenic inflammation, regulation of vascular tone, and neuroprotection, thereby mitigating pathophysiological processes associated with migraine (36, 37). However, although a variety of Chinese patent medicines are available for acute migraine treatment, most are based on other TCM etiological frameworks such as “liver Yang uprising” or “wind-phlegm obstruction” (38). In contrast, SSF is one of the few formulas specifically developed to regulate Ying and Wei. Rooted in the theory of “Ying-Wei disharmony,” SSF integrates five botanicals with documented anti-inflammatory and neuromodulatory properties. Preclinical studies demonstrated SSF’s ability to suppress neurogenic inflammation by modulating CGRP, 5-HT, and NF-κB pathways (19). Additionally, no AEs were observed following the administration of SSF (13). Therefore, there is a critical need for standardized RCTs to validate the efficacy and safety of SSF.

Conducting rigorous RCTs for TCM formulas poses unique challenges, particularly in reconciling TCM’s individualized “syndrome differentiation” approach with standardized trial protocols (39). To address this, our study employs dual diagnostic criteria, specifically the ICHD-3 for migraine diagnosis and a validated “Ying-Wei disharmony” syndrome scale, to ensure precise patient stratification. The selection of outcome measures in this trial aligns with internationally recognized endpoints for acute migraine RCTs (26). The primary outcome, 2-h headache freedom, reflects the gold standard for acute migraine efficacy, capturing the proportion of participants transitioning from moderate/severe pain to complete resolution. This metric is widely endorsed due to its direct clinical relevance, as pain freedom within 2 h signifies rapid therapeutic action, a critical determinant of patient satisfaction and functional recovery. Secondary outcomes further elucidate the multidimensional impact of SSF. The absence of the MBAS at 2 h addresses the heterogeneity of migraine experiences, empowering patients to self-identify their predominant symptom (photophobia, phonophobia, nausea, or vomiting), thereby personalizing efficacy assessment and aligning with patient-centered care paradigms. Sustained relief and freedom from 2 to 24/48 h post-dose evaluate therapeutic durability, distinguishing transient symptom suppression from prolonged efficacy, which serves as key indicators of treatment robustness. Serial VAS scores tracking at predefined intervals (0.5, 1, 2, 4, 24, and 48 h post-dose) provides granular insight into the temporal trajectory of pain reduction, enabling precise characterization of SSF’s onset and maintenance of effect. Additionally, the inclusion of headache recurrence rates and time-to-efficacy onset addresses gaps in conventional trials by quantifying relapse risks and speed of action, both pivotal for clinical decision-making. These outcomes collectively mirror endpoints validated in recent migraine trials (40–42), ensuring methodological rigor and facilitating cross-trial comparisons. This trial also incorporates the TCM-specific syndrome assessment and neuropeptide biomarker analyses to conventional efficacy endpoints, offering a holistic evaluation of SSF’s therapeutic profile. Despite these strengths, limitations warrant consideration. Firstly, the single-dose design focuses on acute attack management but does not address SSF’s long-term prophylactic potential or cumulative effects. Secondly, evaluating headache recurrence rates within 48 h post-dose may underestimate the actual recurrence risk, necessitating extended observation in future studies. Furthermore, by restricting the sample to patients with a specific TCM syndrome, our findings may have limited generalizability to the broader migraine population, particularly in non-TCM clinical settings. This key limitation should be considered when interpreting the results and extrapolating them to other practice contexts.

## 4 Conclusion

In conclusion, this trial will provide robust evidence on the efficacy and safety of SSF for the treatment of acute migraine attacks. The findings, whether demonstrating positive, neutral, or negative outcomes, will hold substantial clinical relevance for migraine management.

## Supplementary material

The Supplementary material for this article can befound online at: https://www.frontiersin.org/articles/10.3389/fneur.2025.1643130/full#supplementary-material

## Glossary

5-HT: 5-Hydroxytryptamine
AE: Adverse event
ALT: Alanine aminotransferase
AST: Aspartate aminotransferase
CGRP: Calcitonin gene-related peptide
CI: Confidence interval
COX-2: Cyclooxygenase-2
Cr: Creatinine
DALY: Disability-adjusted life-year
ECG: Electrocardiogram
ELISA: Enzyme-linked immunosorbent assays
GCP: Good Clinical Practice
HAMA: Hamilton Anxiety Rating Scale
HAMD: Hamilton Depression Rating Scale
HPLC: High-performance liquid chromatography
ICC: Intraclass correlation coefficient
ICHD-3: International Classification of Headache Disorders, 3rd edition
ITMCTR: International Traditional Medicine Clinical Trial Registry
ITT: Intention-to-treat
MAS: Migraine-associated symptom
MBAS: Most bothersome associated symptom
MMRM: Mixed-effects model for repeated measures
NF-κB: Nuclear factor-kappa B
NSAID: Non-steroidal anti-inflammatory drug
PACAP: Pituitary adenylate cyclase-activating polypeptide
PP: Per-protocol
RD: Risk difference
RCT: Randomized controlled trial
RR: Relative risk
SAE: Serious adverse event
SAS: Statistical analysis system
SPIRIT: Standard Protocol Items: Recommendations for Interventional Trials
SS: Safety set
SSF: Sishun Formula
TCM: Traditional Chinese medicine
TG: Trigeminal ganglion
ULN: Upper limit of normal
VAS: Visual Analogue Scale
YWDAS: Ying-Wei Disharmony Assessment Scale
