# Supplementary material for: Sishun Formula for acute migraine attack: study protocol for a double-blind, randomized, placebo-controlled trial
Source: Front Neurol. 2025 Nov 6;16:1643130. doi: 10.3389/fneur.2025.1643130 (PMC12631295; doi:10.3389/fneur.2025.1643130)
Supplement: Supplementary file 1 [file Table_1.pdf]

检 验 报 告 书

文件编号：XLS-QC-MP-0007-OR01(00)

报告书编号： C70C011—24001

**品　　名**	川牛膝配方颗粒	**规　　格**	100g/袋（每1g配方颗粒相当于饮片1.5g）
**批　　号**	24050054	**请验部门**	固体制剂车间
**代 表 量**	14288 袋	**收样日期**	2024年05月16日
**来　　源**	包装工序	**报告日期**	2024年05月31日
**检验依据**	《川牛膝配方颗粒国家药品标准（YBZ-PFKL-2021027）》		

**检验项目**	**检验标准**	**检验结果**
**性　状**	本品应为黄色至黄棕色颗粒；气微，味甜。	符合规定
**薄层鉴别**	供试品色谱中，在与川牛膝对照药材色谱和杯苋甾酮对照品色谱相应的位置上，应显相同颜色的荧光斑点。	符合规定
**特征图谱**	应符合规定	符合规定
**检　查**		
粒　度	不能通过一号筛与能通过五号筛的总和应≤15%	2.0%
水　分	≤8.0%	1.9%
溶化性	应全部溶化或轻微浑浊，不得有焦屑或异物	符合规定
装　量	平均装量应≥100g，每袋装量应≥97g	符合规定
**浸出物**	乙醇热浸出物≥15.0%	22.9%
**含量测定**	杯苋甾酮应为0.45mg/g～1.50mg/g	0.76mg/g
**微生物限度检查**		
需氧菌总数	≤10^3 CFU/克（最大可接受2000 CFU/克）	1.0×10^2 CFU/克
霉菌和酵母菌总数	≤10^2 CFU/克（最大可接受200 CFU/克）	55 CFU/克
大肠埃希菌	不得检出	未检出

以下空白

结论：本品按《川牛膝配方颗粒国家药品标准（YBZ-PFKL-2021027）》检验，结果符合规定。

（印章：四川新绿色药业科技发展有限公司 质检专用章）

批准人：[签名] 2024.05.31　　复核人：[签名] 2024.05.31　　编制人：[签名] 2024.05.31

声明：1、本报告检验结果仅对送检样品负责；
2、对本报告若有异议，应于收到报告之日起七个工作日内向本中心提出，逾期将自动视为承认本检测报告。

检测中心地址：四川省彭州市致和镇东河东路279号　　电话号码： 028-83888888

检 验 报 告 书

文件编号：XLS-QC-MP-0007-OR01(00)

报告书编号： C70B003—24007

**品　　名**	白芍配方颗粒	**规　　格**	100g/袋（每1g配方颗粒相当于饮片4.5g）
**批　　号**	24060193	**请验部门**	固体制剂车间
**代 表 量**	20589 袋	**收样日期**	2024年06月23日
**来　　源**	包装工序	**报告日期**	2024年07月14日
**检验依据**	《白芍配方颗粒国家药品标准（YBZ-PFKL-2021002）》		

**检验项目**	**检验标准**	**检验结果**
**性　状**	本品应为黄白色至黄棕色的颗粒；气微，味苦、微酸。	符合规定
**薄层鉴别**	供试品色谱中，在与白芍对照药材和芍药苷对照品色谱相应的位置上，应显相同颜色的斑点。	符合规定
**特征图谱**	应符合规定	符合规定
**检　查**		
粒　度	不能通过一号筛与能通过五号筛的总和应≤15%	3.0%
水　分	≤8.0%	3.8%
溶化性	应全部溶化或轻微浑浊，不得有焦屑或异物	符合规定
装　量	平均装量应≥100g，每袋装量应≥97g	符合规定
硫熏检查	应符合规定	符合规定
**浸出物**	乙醇热浸出物≥35.0%	58.9%
**含量测定**	芍药苷应为65.0mg/g～137.0mg/g	89.3mg/g
重金属及有害元素（*）	铅≤5mg/kg	0.06mg/kg
	镉≤1mg/kg	低于0.0071mg/kg
	砷≤2mg/kg	0.3mg/kg
	汞≤0.2mg/kg	低于0.01673mg/kg
	铜≤20mg/kg	4.5mg/kg
**微生物限度检查**		
需氧菌总数	≤10^3 CFU/克（最大可接受2000 CFU/克）	<20 CFU/克
霉菌和酵母菌总数	≤10^2 CFU/克（最大可接受200 CFU/克）	30 CFU/克
大肠埃希菌	不得检出	未检出

备注：带（*）项目结果为引用结果。

备注：重金属 检测
ICP-MC检出限（单位mg/kg）：Pb 0.04197　Cd 0.0071　As 0.01279　Hg 0.01673　Cu 0.03591
当检测结果低于上述值时以低于上述数值出具报告

以下空白

结论：本品按《白芍配方颗粒国家药品标准（YBZ-PFKL-2021002）》检验，结果符合规定。

四川新绿色药业科技发展有限公司 质检专用章

批准人：[signature] 2024.07.14　　复核人：[signature] 2024.07.14　　编制人：[signature] 2024.07.14

声明：1、本报告检验结果仅对送检样品负责；
2、对本报告若有异议，应于收到报告之日起七个工作日内向本中心提出，逾期将自动视为承认本检测报告。
检测中心地址：四川省彭州市致和镇东河东路279号　　电话号码：028-83888888

检 验 报 告 书

文件编号：XLS-QC-MP-0007-OR01(00)

报告书编号： C70C006—24003

**品　　名**	川芎配方颗粒	**规　　格**	100g/袋（每1g配方颗粒相当于饮片3g）
**批　　号**	24060211	**请验部门**	固体制剂车间
**代 表 量**	19394 袋	**收样日期**	2024年06月20日
**来　　源**	包装工序	**报告日期**	2024年06月28日
**检验依据**	《川芎配方颗粒国家药品标准（YBZ-PFKL-2021029）》		

**检验项目**	**检验标准**	**检验结果**
**性　状**	本品应为淡黄色至黄棕色的颗粒；气微香，味微苦、辛。	符合规定
**薄层鉴别**	供试品色谱中，在与川芎对照药材和阿魏酸对照品色谱相应的位置上，应显相同颜色的荧光斑点。	符合规定
**特征图谱**	应符合规定	符合规定
**检　查**		
粒　度	不能通过一号筛与能通过五号筛的总和应≤15%	3.6%
水　分	≤8.0%	4.1%
溶化性	应全部溶化或轻微浑浊，不得有焦屑或异物	符合规定
装　量	平均装量应≥100g，每袋装量应≥97g	符合规定
**浸出物**	乙醇热浸出物≥15.0%	30.6%
**含量测定**	阿魏酸应为1.5mg/g～4.5mg/g	1.9mg/g
**微生物限度检查**		
需氧菌总数	≤10^3 CFU/克（最大可接受2000 CFU/克）	10 CFU/克
霉菌和酵母菌总数	≤10^2 CFU/克（最大可接受200 CFU/克）	20 CFU/克
大肠埃希菌	不得检出	未检出

以下空白

结论：本品按《川芎配方颗粒国家药品标准（YBZ-PFKL-2021029）》检验，结果符合规定。

四川新绿色药业科技发展有限公司 质检专用章

批准人：[signature] 2024.06.28　　复核人：[signature] 2024.06.28　　编制人：[signature] 2024.06.28

声明：1、本报告检验结果仅对送检样品负责；
2、对本报告若有异议，应于收到报告之日起七个工作日内向本中心提出，逾期将自动视为承认本检测报告。

检测中心地址：四川省彭州市致和镇东河东路279号　　电话号码： 028-83888888

检 验 报 告 书

文件编号：XLS-QC-MP-0007-OR01(00)

报告书编号： C70H045—24004

**品　　名**	红景天配方颗粒	**规　　格**	100g/袋（每1g配方颗粒相当于饮片3g）
**批　　号**	24070034	**请验部门**	固体制剂车间
**代 表 量**	4796 袋	**收样日期**	2024年07月04日
**来　　源**	包装工序	**报告日期**	2024年07月25日
**检验依据**	《红景天配方颗粒国家药品标准（YBZ-PFKL-2022023）》		

**检验项目**	**检验标准**	**检验结果**
**性　状**	本品应为浅黄棕色至红棕色的颗粒；气微，味微苦涩。	符合规定
**薄层鉴别**	供试品色谱中，在与红景天对照药材色谱相应的位置上，应显相同颜色的斑点。	符合规定
**特征图谱**	应符合规定	符合规定
**检　查**		
粒　度	不能通过一号筛与能通过五号筛的总和应≤15%	2.8%
水　分	≤8.0%	3.6%
溶化性	应全部溶化或轻微浑浊，不得有焦屑或异物	符合规定
装　量	平均装量应≥100g，每袋装量应≥97g	符合规定
**浸出物**	乙醇热浸出物≥30.0%	54.1%
**含量测定**	红景天苷应为9.0mg/g～40.0mg/g	14.0mg/g
	没食子酸应为16.0mg/g～54.0mg/g	34.1mg/g
**微生物限度检查**		
需氧菌总数	≤10^3 CFU/克（最大可接受2000 CFU/克）	10 CFU/克
霉菌和酵母菌总数	≤10^2 CFU/克（最大可接受200 CFU/克）	10 CFU/克
大肠埃希菌	不得检出	未检出

以下空白

结论：本品按《红景天配方颗粒国家药品标准（YBZ-PFKL-2022023）》检验，结果符合规定。

四川新绿色药业科技发展有限公司 质检专用章

批准人：［签名］2024.07.25　　复核人：［签名］2024.07.25　　编制人：［签名］2024.07.25

声明：1、本报告检验结果仅对送检样品负责；
2、对本报告若有异议，应于收到报告之日起七个工作日内向本中心提出，逾期将自动视为承认本检测报告。

检测中心地址：四川省彭州市致和镇东河东路279号　　电话号码： 028-83888888

检 验 报 告 书

文件编号：XLS-QC-MP-0007-OR01(00)

报告书编号： C90G005—24006

**品　　名**	桂枝配方颗粒	**规　　格**	100g/袋（每1g配方颗粒相当于饮片7.5g）
**批　　号**	24070109	**请验部门**	固体制剂车间
**代 表 量**	3855 袋	**收样日期**	2024年07月17日
**来　　源**	包装工序	**报告日期**	2024年07月22日
**检验依据**	《桂枝配方颗粒（SCYPBZ（PFKL）-2021242）》		

**检验项目**	**检验标准**	**检验结果**
**性　状**	本品应为灰棕色至红棕色的颗粒；有特异香气，味甜、微苦、微辛。	符合规定
**薄层鉴别**	供试品色谱中，在与桂枝对照药材色谱和肉桂酸对照品、桂皮醛对照品色谱相应的位置上，应显相同颜色的斑点。	符合规定
**特征图谱**	应符合规定	符合规定
**检　查**		
粒　度	不能通过一号筛与能通过五号筛的总和应≤15%	6.0%
水　分	≤8.0%	2.2%
溶化性	应全部溶化或轻微浑浊，不得有焦屑或异物	符合规定
装　量	平均装量应≥100g，每袋装量应≥97g	符合规定
**浸出物**	乙醇热浸出物≥18.0%	34.0%
**含量测定**	桂皮醛应为6.5mg/g～18.0mg/g	6.9mg/g
**微生物限度检查**		
需氧菌总数	≤10^3 CFU/克（最大可接受2000 CFU/克）	2.1×10^2 CFU/克
霉菌和酵母菌总数	≤10^2 CFU/克（最大可接受200 CFU/克）	<20 CFU/克
大肠埃希菌	不得检出	未检出

以下空白

结论：本品按《桂枝配方颗粒（SCYPBZ（PFKL）-2021242）》检验，结果符合规定。

批准人：[illegible] 2024.07.22　　复核人：[illegible] 2024.07.22　　编制人：[illegible] 2024.07.22

声明：1、本报告检验结果仅对送检样品负责；
2、对本报告若有异议，应于收到报告之日起七个工作日内向本中心提出，逾期将自动视为承认本检测报告。

检测中心地址：四川省彭州市致和镇东河东路279号　　电话号码： 028-83888888
